# Asthma management in British South Asian children: an application of the candidacy framework to a qualitative understanding of barriers to effective and accessible asthma care

**DOI:** 10.1186/s12889-016-3181-z

**Published:** 2016-06-14

**Authors:** Nicky Hudson, Lorraine Culley, Mark Johnson, Melanie McFeeters, Noelle Robertson, Emma Angell, Monica Lakhanpaul

**Affiliations:** School of Applied Social Sciences, De Montfort University, Hawthorn Building, The Gateway, Leicester, LE1 9BH UK; Mary Seacole Research Centre, De Montfort University, Leicester, UK; NHS England, Leicester, UK; School of Psychology, University of Leicester, Leicester, UK; SAPPHIRE Research Group, University of Leicester, Leicester, UK; Institute of Child Health, University College London, London, UK

**Keywords:** Chronic illness, Young people, Healthcare, Minority ethnic, Service delivery, NHS, Long-term conditions

## Abstract

**Background:**

In the UK, people of South Asian origin with asthma experience excess morbidity, with hospitalisation rates three times those of the majority White population and evidence suggests that South Asian children with asthma are more likely to suffer uncontrolled symptoms and hospital admissions with acute asthma compared to White British children. This paper draws on data from The Management and Interventions for Asthma (MIA) study to identify the operation of barriers to optimal care and good asthma control for South Asian children.

**Methods:**

The MIA study followed a multi-phase, iterative, participatory design, underpinned by the socio-ecological model. Findings presented here are from face-to face, semi-structured interviews with South Asian (Indian, Pakistani and Bangladeshi origin) parents and carers of a child with asthma (*n* = 49). Interviews were conducted in English or relevant South Asian languages using specially trained community facilitators. Data were transcribed verbatim and analysed according to the principles of interpretive thematic analysis, facilitated by the use of NVivo.

**Results:**

Seven dimensions of candidacy are identified: identification of candidacy; navigation; the permeability of asthma services; appearances at health services; adjudications; offers and resistance and operating conditions in the local production of candidacy. The analysis demonstrates several ways in which a potential lack of alignment between the priorities and competencies of British South Asian families and the organization of health services combine to create vulnerabilities and difficulties in effectively managing childhood asthma.

**Conclusions:**

Healthcare systems have a responsibility to develop services that are sensitive and appropriate to the needs of their communities. In South Asian communities, further efforts are required to raise awareness of symptoms and effectively communicate how, when and where to seek help for children. There is a need for improved diagnosis and consistent, effectively communicated information, especially regarding medication. Parents made several suggestions for improving services: presentations about asthma at easily accessible community venues; an advice centre or telephone helpline to answer queries; opportunities for sharing experiences with other families; having information provided in South Asian languages; longer GP appointments; extended use of asthma nurses; and better education for healthcare professionals to ensure consistency of care and advice.

## Background

The outcomes for children with long-term disease in the UK are amongst some of the worst in Europe. Childhood asthma is one of the most common long-term conditions of childhood, and in the UK, current prevalence estimates suggest that 1.1 million children, or 1 in 11 children, will experience asthma at some point in their childhood [1]. Every year 44,000 children are admitted to hospital in the UK with between 40 and 50 children dying as a result of their condition [1], thus, asthma places a substantial burden of care on families, communities and healthcare services [1]. The total cost of asthma to the UK economy, including the use of primary and secondary care services, emergency services, medications and lost productivity from days off work/school is estimated to be over £2.3 billion per year [2].

Minority ethnic groups frequently experience poorer outcomes for long-term conditions, and experience higher morbidity and mortality rates than majority populations [3, 4]. In the UK, people of South Asian origin with asthma experience excess morbidity, with hospitalisation rates three times those of the majority White population [5] and evidence suggests that South Asian children with asthma are more likely to suffer uncontrolled symptoms and hospital admissions with acute asthma compared to White British children independent of objective disease severity [5–7]. The 2010 White Paper ‘Equity and Excellence’ focused on the need to address ethnicity and inequalities in health [8] with the Marmot Review [9] identifying numerous influences on health inequalities and the urgent need to address them. Reducing ethnic inequalities in health outcomes has been a major policy goal for successive UK governments [10].

A recent literature review of the barriers and facilitators to asthma management in South Asian children carried out by the authors identified barriers to care including: understandings regarding the nature of asthma; under-use of preventer medications (due to under-prescription and fears of over-use); non-acceptance and denial of asthma; reliance on emergency departments in preference to primary care; difficulties in perception of symptom severity; language barriers, and use of complementary therapies [11]. The review highlighted the importance of not only establishing the nature of a barrier for minority groups, but also its origins, to improve outcomes for children’s health.

The Management and Interventions for Asthma (MIA) study explored the experiences of British South Asian families who had a child (or children) with asthma in order to gather evidence about barriers to optimal asthma care. The study was designed to develop an intervention-planning framework for South Asian children with asthma with a central objective of exploring perceptions, understandings and experiences of care [12]. This paper draws on one major aspect of that study: qualitative interviews with parents/carers of children with asthma to illustrate the barriers to optimal care and good asthma control that families experienced. It draws on data from South Asian parents and carers (hereafter ‘parents’) in order to demonstrate specific barriers that were experienced in accessing care, and using the ‘candidacy’ framework [13], it explores the ways in which barriers to care manifested in different contexts.

### The candidacy framework

The candidacy framework emphasises the complex and contingent character of health-care access and therefore provides a critical conceptual lens through which to interpret our findings and to explore the social patterning of perceptions of asthma and health services. Candidacy describes the negotiation of eligibility for medical treatment and care between individuals and healthcare services. Dixon-Woods et al. define this as ‘a dynamic and contingent process, constantly being defined and redefined through interactions between individuals and professionals, including how “cases” are constructed’ ([13], p7). This framework was developed as a counter to existing notions of ‘access’, which draw on data about utilisation but which often fail to capture the complex processes involved in navigating care. The Candidacy framework offers a means of understanding the complex negotiations that users of healthcare settings have to engage in because of a potential misalignment between individual priorities and needs, and the organisation of healthcare services. It takes into account the context and the setting of these interactions together with the social patterning of perceptions of health and health services, and explores how these complex negotiations may act as barriers to the receipt of care. The framework is constituted by seven dimensions: identification of candidacy; navigation; the permeability of services; appearances at health services; adjudications; offers and resistance and operating conditions and the local production of candidacy (Table 1). The MIA study was designed to explore the perceptions of asthma in South Asian communities, alongside an exploration of the challenges which face South Asian parents in navigating healthcare systems [12]. We draw on examples from the MIA study data to explore in detail some of the barriers to asthma care for British South Asian families.

**Table 1:** The Dimensions of candidacy

Candidacy dimension	Definition	Examples in MIA study
Identification of candidacy	The ability of individuals to recognise their symptoms when they first manifest as requiring medical assistance or intervention	• Families’ perceptions of asthma • Parents’ recognition of asthma symptoms
Navigation	The need for knowledge about services and the ability to mobilise a range of resources (practical, financial) that enable access to services	• Awareness about asthma services • Time off work for appointments
Permeability	The ease with which people can gain access to different healthcare services	• GP practices as permeable • Practice nurses as assisting access • Need for negotiation of language differences
Appearances at health services	The assertion of a claim to candidacy for medical attention or intervention by appearing at a service	• Getting a diagnosis • Managing emergency situations (attacks) • On-going management
Adjudications	The judgements and decisions made by professionals, which allow or inhibit continued progression of candidacy	• Inconsistent management and unclear or contradictory advice given by different health professionals
Offers and resistance	The offer of care or treatment made my services, and the resistance to those offers by users	• Refusal of annual reviews • Non-use of asthma medication
Operating conditions and the local production of candidacy	Local or context-specific influences on interactions between patients and practitioners	• The uncertain and contingent nature of asthma • Local South Asian community networks

## Methods

The MIA study followed a multi-phase, iterative, participatory research design, underpinned by the socio-ecological model [14]. In the main data collection phase of the study, semi-structured interviews were carried out with parents of, and children with asthma in order to explore the experiences of living with asthma, and as we describe below, their experiences of accessing care. Purposive sampling was used to ensure proportional representation from each of the six main UK-based South Asian religious-ethnic groups: Indian Gujarati Hindu; Indian Gujarati Muslim; Pakistani Muslim; Bangladeshi Muslim; Indian Punjabi Sikh and Indian Punjabi Hindu. This also ensured inclusion of children with a broad range of asthma severity (judged by position on the BTS (British Thoracic Society) steps of asthma management) [15] and from both genders. Families were recruited face-to-face, via telephone and by mail via a number of routes including: GP practices; Accident and Emergency; specialist asthma clinics; paediatric clinics; community events; and word of mouth/snowballing.

Research protocols were sensitive to the needs of participants throughout and ensured that distress was minimised and anonymity and confidentiality were safeguarded [12]. Participant information and consent forms were provided either in English written format or in translated audio format. Where study materials were provided in English written format, consent was taken in writing in English unless a participant did not have sufficient literacy skills, in which case verbal audio-recorded consent was used. Where study materials were provided in translated audio format, the consent form was read out by the community facilitator in the relevant language and the participant was asked to confirm that they understood and agreed with each point. This process was recorded. If they wished, participants could also initial and sign the written consent form at the same time. A copy of the audio-recorded consent was given to the participant to take away with them (for a full description, see 12).

Interviews were carried out in families’ homes either in English by the female research fellow (a qualified paediatrician, trained in research methods and not previously known to the participants or involved in their care), or in relevant South Asian languages with the assistance of specially trained community facilitators. These interviews were later transcribed into English [16]. Interview schedules were developed with reference to the literature, and in partnership between the research team and community facilitators. Questions were piloted and covered the following topics: understandings of asthma; family and community perceptions of asthma; day to day management; medical management; interactions with healthcare providers and the quality of healthcare services and provision. Interviews lasted an average of one to two hours. Data were digitally recorded, transcribed verbatim and analysed according to the principles of interpretive thematic analysis and facilitated by the use of NVivo [17]. Following an initial analysis of data, the findings were presented to South Asian family participants in a workshop, to validate the interpretation and to ask families to rank the issues identified in the interviews. (For a full description of the analysis methods, see [12]) For the purposes of this paper, further qualitative analysis was conducted by two authors (NH and LC), using a process of ‘charting’, in which themes from the original thematic analysis were populated into a table of candidacy constructs.

## Results

### Profile of the sample

A total of 81 expressions of interest were received from volunteers enquiring about participation in an interview. Eight were ineligible to participate and 35 were not recruited for other reasons (such as because they could not be reached on follow up, they were no longer interested, or because a suitable time for interview could not be found). The remaining 38 expressions of interest resulted in interviews with 49 members of thirty family ‘cases’. This included 44 parents (29 mothers; 15 fathers) and five secondary carers^1^ (Table 2). There were also 33 South Asian children recruited in to the study (20 boys, 13 girls), aged between 5 and 12 years old, seven at BTS level 1, 17 at BTS level 2, six at BTS level 3, three at BTS level 4 and no children at BTS level 5.^2^ This paper is based on the data from the adult parents and carers.^3^

**Table 2:** Ethnicity, religion and parent/carer status of participants

	Indian Gujarati			Pakistani	Bangladeshi	Indian Punjabi	
Religion	Hindu	Muslim	Jain	Muslim	Muslim	Sikh	Hindu
Mothers	6	4	1	4	7	6	1
Fathers	4	3	1	2	1	3	1
Secondary carers	0	0	0	1	1	3	0
Total	10	7	2	7	9	12	2

## Results and discussion: Candidacy and childhood asthma

### Identification of candidacy

This dimension of the candidacy framework relates to the ability of individuals to recognize their symptoms when they first manifest as requiring medical assistance or intervention and is significant in determining if or how they will proceed to assert a claim to candidacy and therefore to seek treatment or care.

In the MIA study identification of candidacy related to ways in which parents perceived asthma and recognised symptoms their child was experiencing as being those of asthma. Previous knowledge of asthma appeared important both to recognise symptoms and to subsequently manage asthma. Knowledge about asthma and its management appeared more likely if participants already had someone in the family with asthma (sometimes one or both of the child’s parents or older siblings). However, in the absence of this, parents were unlikely to describe having heard about asthma.

*“And because we’ve never really been exposed to something like asthma within the, within our immediate families, neither my wife or myself, it was something which was slightly unusual for us, we didn’t probably recognise it as an onset… I’d say, if anything, we were quite ignorant of the condition itself, and had no previous knowledge or of managing or living with people with asthma ourselves, so I think, we took it very much as okay, maybe this is just a small ongoing thing, he’s quite young, he’ll grow out of it, whatever it is.”* (Father, Pakistani)

Approximately a third of parents specifically stated that they felt unclear about the origins of asthma. Aetiological ‘causes’ of asthma and exacerbations or ‘triggers’ for asthmatic symptoms however, were often conflated in the interviews. The most commonly mentioned causes were attributed to environmental conditions (e.g. the weather, damp, dust, pollen, pollution) and physiological or genetic causes (e.g. weak immune system, asthma being hereditary, not breast feeding). In addition, causes including dietary and lifestyle factors were mentioned as were ideas that asthma was pre-destined or fated for a child. Of the 18 South Asian parents who discussed religion and pre-destination, 10 made explicit causal attributions. A third of parents felt that the cause of asthma was either not known or could not be identified. Parents reported that advice was often given by relatives and close friends (though not always adhered to). In some families, grandparents lived with the family, and where this was the case they had a greater role to play in advising and decision-making around the health of the child with asthma:

*‘…you have this extended influence around you. There’s no doubt that clearly, living in the UK and obviously, born and raised here, that you’re far more accepting of different opinions. However, because you really do interfere quite heavily with an extended family… so it’s not unusual for the family to give advice because they’ll see it as, oh yeah, you know, we just got, it’s one of their own, it’s not unusual”.* (Father, Pakistani)

The particular features of asthma as a chronic but uncertain and somewhat ambiguous condition of unknown aetiology impacted on parents’ ability to identify a claim to candidacy. All parents in this study had previously asserted a claim to candidacy, having at some point seen a healthcare professional about their child’s symptoms, despite this uncertain context. Yet there was a great deal of variability in the success people had in getting a formal diagnosis on the basis of these ensuing claims to candidacy and there were differences in the ways in which these claims were asserted via health services. Getting a diagnosis was identified as a major problem (as discussed in the following sections).

### Navigation

Using healthcare services requires the mobilization of knowledge and resources [18]. Dixon-Woods et al. draw attention to the possibility that the need for practical resources, such as transport or flexible working arrangements, may particularly impact the ability of those from vulnerable groups to access healthcare, and that members of these groups may also be less aware of the services available. More recently, the concept of navigation has been used to explain the experiences of migrants when accessing complex healthcare systems in Europe [19]. In our study, most parents described being aware that the GP (general practitioner) was the first point of access for routine asthma care, though this was often more complex than they had anticipated. In emergency situations, however, there was more variability in how services were navigated.

A small number of parents described practical difficulties in managing their child’s asthma-related healthcare, in terms of finding relevant information about the care available and being able to manage time off work in order to attend appointments:

*“we both worked at the same place so it was really hard …because of her going to hospital and being ill, all our annual leave’s been eaten up like that and we can’t have like a family holiday together because we go to work at one place.”* (Mother, Indian Punjabi)

*“I wish I could ask someone. There’s no one. Myself. Sometime I just get the inhaler leaflet out, the instructions, read it over and over and over but still, you know, like, it’s same thing you reading it, like to finally satisfy, I think, if I read it again, there might be something I missed out.”* (Mother, Bangladeshi)

### The permeability of services

Dixon-Woods et al. use the construct of “permeability” to refer to the ease with which people can gain access to healthcare services. They offer the example of accident and emergency departments to highlight services which are more ‘porous’ and require fewer resources for access, in contrast to those, for example, which may require users to be more culturally and linguistically familiar with the organisational structures and processes of the NHS, such as referral and attendance at pre-arranged out patient appointments. In our study, parents felt that GPs were relatively permeable for routine appointments once a diagnosis had been given. This permeability was enhanced for some parents who shared cultural or linguistic characteristics with their GP [20].

Practice nurses dedicated to asthma care were felt to provide ‘navigational assistance’ [19] for parents who were otherwise uncertain about how to manage their child’s asthma, since they often uniquely had more dedicated time and focus than GPs to spend with a family.

*“And she actually talks to you and asks him to show her exactly how he uses his inhalers and the spacer as well… The practical side; and how many seconds to wait between puffs and… So he actually has to do it in front of her so she knows he’s going it properly…. I think the asthma nurse is vital.”* (Mother, Indian Punjabi)

However, not all families were accessing this nurse-led service (see below: appearing at health services). There was little discussion in the interviews about referrals to specialist tertiary care; few participants described having been referred, a finding which may be related to a lack of permeability, but may also be a reflection of the management of asthma in primary care and the severity of asthma amongst the majority of families in this study.

Language barriers were not directly raised as a major barrier to access by parents, but the data suggest that this may be because participants had developed their own mechanisms to overcome these. Parents reported that they used strategies such as seeking out GPs who could speak the same language, choosing to attend the emergency department or accessing a local pharmacist who was often of the same cultural background or could offer advice in a language other than English.

*“We try to go and see our Punjabi speaking doctor because we can understand what he is trying to tell us, but we have seen a white lady doctor as well, she is very nice but with her, we need someone’s help to get the information”.* (Secondary carer, female, British Punjabi)

*“You see [my wife] want more information, probably haven’t got time or something so that’s why she went straight to the pharmacist…the pharmacist says the same language she speaks”* (Father, Indian Gujarati)

### Appearances at health services

This dimension of the framework requires that people assert their claim to candidacy: i.e. they make a case for help or intervention by voicing their needs and presenting demands in relation to healthcare. Dixon-Woods et al. highlight that this requirement places some groups at greater risk of vulnerability, since they may not have the required competencies to successfully enact candidacy, due to a greater social distance between themselves and the professionals whom they encounter. Data from this study demonstrated that for parents with a child with asthma, there were three key ‘moments’ when they might appear at services within the NHS. These were:

1. During the process of getting a diagnosis (mostly with the GP but in some cases with emergency department staff)
2. When the child had an acute asthma attack (for example at NHS Direct, Out of Hours service or an emergency department) and;
3. During ongoing management (most likely to be the practice nurse, or in some cases, a pharmacist)

Getting a diagnosis for asthma was disclosed as a difficult experience with considerable negotiation described in relation to this. Thirteen of 30 families reported that a direct diagnosis of asthma had not been conveyed, despite the fact that all participants recruited in to the study had been prescribed asthma medicines. Participants described the following problems in getting a diagnosis: undue delays in the process; feeling ‘fobbed off’ or not being taken seriously; repeat visits to the GP with recurring problems; and feeling that healthcare professionals were reluctant to make a diagnosis. Not receiving a diagnosis led to feelings of frustration, upset and anger and suggests that at this point, services may be experienced as less ‘permeable’.

*“Every time there is a different doctor! Every time different medicine! They cannot reach to a conclusion or there is no solid result or a procedure to follow… We were given various appointments and at every stage we had to wait for results. For example he had a blood test and then wait two weeks for the results. In the meantime if he had difficulties we just had to keep giving him medication. In short there was no quick diagnosis. Too late.”* (Mother, Indian Gujarati)

There was a perception amongst participants that at symptom onset (and prior to diagnosis), healthcare professionals (usually the GP) did not take their concerns sufficiently seriously or were reluctant to diagnose their child with asthma, and as a result, their claims for candidacy were felt to be unsuccessful (see below, ‘adjudications’). Not having a diagnosis for their child’s symptoms led to a great deal of uncertainty about how to proceed. Without an official ‘label’ of asthma, parents were unclear about whether to give medicines or how to manage symptoms. Whether or not they successfully received a diagnosis also impacted on their future decision-making about appearing at healthcare services.

On receiving a diagnosis for their child, a number of parents reported relief and for some, vindication of their experiences. They felt that this conferred a status enabling them to make further claims to candidacy, notably access to review appointments and asthma medications, in addition to easing communication about the condition with their child and with others, such as the child’s school.

*“Well the difference is that if; if you have got asthma then I can say that my child has got asthma. I mean if somebody ask me, I just say no, no, no the doctor hasn’t diagnosed it as asthma, I always say that”.* (Mother, Indian Gujarati)

In some cases a diagnosis had been made or communicated by hospital staff following an acute attack during an emergency visit to hospital. For those parents, this had been a defining point in the process and in some cases this was interpreted as meaning that the staff at the hospital had more expertise and knowledge about asthma than the GP.

A significant proportion of families had experienced the need to access the NHS in an emergency or non-routine situation. This had involved decisions by the parents about two things: 1) the unusualness and severity of the child’s symptoms, and 2) where to seek help. The general perception was that the GP was the first point of contact in managing their child’s asthma, where appropriate. However, when symptoms were perceived to be beyond the assistance or expertise of the GP, or if the practice was closed during evenings and weekends, parents needed to make a decision about where to ‘appear’ at healthcare services. A higher proportion of parents described being likely to self-refer to the emergency department (ED) in this situation (13 of 30 families), than to access their own doctor’s ‘out of hours’ service (3 of 30). A small number of parents reported having been worried enough to call an ambulance. The data suggest several possible reasons for higher levels of attendance at ED, notably previous experiences of using the health system, e.g. being referred to ED during previous acute attacks, or experiencing negative events or delayed appointments with their GP. However, it was sometimes a consequence of difficulties and delay in recognising the severity of symptoms until ED attendance became the only option, because symptoms had escalated to severe levels. None of the parents had any formal means of assessing their child’s symptoms. None recalled being provided with a written asthma plan and little use of peak flow meters was reported.

Ongoing management of a child’s asthma often required that parents interacted with healthcare professionals regularly, usually via appearance at the regular or annual asthma review with the practice nurse. Most parents who described attending reviews (approximately a third) were satisfied with this process. Here, the review was seen as a process which involved ‘checking’ things like inhaler technique and peak flow rather than about gaining new information or for changes in medicines. As suggested above, the nurses who provided this service were viewed positively for the navigational role that they provided. Amongst the families who were not accessing the annual review, this was often either because they considered it of little value or because they were seeking advice from other sources, such as pharmacists (see below). Almost a third of parents specifically described accessing the pharmacist for advice about a child’s asthma and medications. Sometimes this was for a second opinion, sometimes for additional information about the medicines and in some cases for demonstration of inhaler or spacer use. Pharmacists therefore played an important navigational role for the management of asthma in South Asian families.

### Adjudications

‘Adjudications’ are the judgements and decisions made by professionals, which allow or inhibit continued progression of candidacy. Other studies have demonstrated that the role of healthcare professionals can be crucial in determining successful access to treatment or further healthcare, such as referral to specialist services [21]. In this study, parents were especially vulnerable to adjudications about their child’s health in the process of seeking an asthma diagnosis. The uncertainty associated with asthma meant that unclear decisions about management, and ambiguous advice about symptoms from healthcare professionals, could have a negative effect on experiences. There were numerous descriptions in the interviews from parents who felt that they were left in ‘limbo’ in relation to their child’s healthcare needs, particularly in the absence of a definitive diagnosis.

*“Now we, that’s the funny thing. I just don’t know if I’m coming or going with them. I want to know if she has got it. If she has, then, is it a permanent thing? Or is she going to get out the phase? So, when I did ask, he goes that ‘With kids, you can’t tell’. And I go, ‘What is the cause of it’? He goes ‘It could be many stuff’. He’s not given me a full diagnosis. He hasn’t told me, he hasn’t even confirmed she has got asthma.”* (Mother, Bangladeshi)

The absence of clear guidance was associated with parents’ strategies to deal with their children’s symptoms including attempts to self-manage with over the counter or alternative medicines, seeking second opinions or regular, repeat visits to the GP or ED. While most parents reported many positive experiences with health professionals, especially asthma nurses, several also spoke of inconsistent management and unclear or contradictory advice given by different health professionals which often lead to confusion about different medications available and the techniques required to deliver them. Many referred to having inadequate explanations about asthma and insufficient information about how to manage it.

### Offers and resistance

Dixon-Woods et al. suggest that much of the previous work on healthcare utilisation assumes (explicitly or implicitly) that non-utilisation is a direct result of the absence of an offer. Instead, they suggest that we need to better understand how people may refuse offers of care or treatment, for example refusal of referral or of medication, since this will give a fuller insight into the complexity of issues of utilisation and access. In the MIA study this manifested as the refusal or non-use of annual asthma reviews, and the non-use of asthma medicines.

As highlighted above, a number of families reported that they chose not to access the annual asthma reviews on offer to them via their GP practice. Of those discussing their reasons for non-attendance, they suggested that they felt the reviews were not hugely helpful, and in many cases this was coupled with the fact that parents were seeking guidance and information about their child’s asthma elsewhere. This often involved advice from the local pharmacist, in this example, because a mis-match in language created uncertainty between the parent and primary care staff.

*“You see [my wife] want more information… so that’s why she went straight to the pharmacist… The pharmacist [speaks] the same language she speaks… so that’s why sometimes she don’t understand what doctor says. […] She still don’t believe in the doctor, she will believe in doctor but not that much, not 100% like 90% so she will always go for second opinion to the pharmacist.”* (Father, Indian Gujarati)

In a second example of resistance, making decisions about when and how to use asthma medicines was an issue that a significant proportion of families in this study discussed (26 of 30). This related to participants’ knowledge and beliefs about both asthma and the effectiveness of the medicine. Asthma medicines were described as not being used consistently and this appeared to relate to the issue of diagnosis: if a child had not received a firm diagnosis, parents expressed more reluctance or resistance, to giving medicines. Parents gave a number of reasons why they varied in adherence to their medication. Reasons for increasing the dosage mainly centred on instances of viruses and colds. Reasons for not adhering to the medicines were: a lack of diagnosis; concerns about side effects; an absence of symptoms at certain times of the year; children refusing or deciding not to take their medicine; and misunderstandings about how to take the medicine.

Perceived side-effects from asthma medicines were commonly discussed (22 of 30 families) and included: concerns about children’s growth; stomach problems; heart problems; addiction; reduced immunity; oral thrush; mood or behavioural issues; and reduced immunity to asthma medicines. One parent believed that asthma medicines made their child’s symptoms worse. Concerns about long-term use of steroid use, particularly in relation to dependence caused by ‘overreliance’, was also a major concern for several parents. The data suggest that possible side-effects had not always been explained to people effectively enough and this in turn related to assumptions being made by parents and the decisions they made about their child using medications.

*“I didn’t think it was right. I’ve heard (…) body gets used to steroid, it’s not good. Then nothing works on you.”* (Mother, Indian Gujarati)

### Operating conditions and the local production of candidacy

In the final dimension of the candidacy framework, Dixon-Woods et al. suggest that local factors can have an impact on the production of candidacy. They suggest that ‘these are the contingent and locally specific influences on interactions between practitioners and patients, which may be emergent over time through repeated encounters’ ([13], p 8). We have interpreted ‘local’ in two ways in this paper. Our data suggest that at least in part, these ‘local’ or ‘context specific’ factors related to the disease characteristics of asthma as an ambiguous, uncertain condition about which parents tended to have little knowledge and to which healthcare professionals’ responses are often ambivalent. A second feature of this particular study related to geographical locality, whereby the research was conducted within a well-established local South Asian community and further characterised by the existence of a number of GP practices and pharmacists with cultural and linguistic knowledge aligned to some of their South Asian patients. Whilst local linguistic diversity was less of a concern for the parents who spoke good English, for those for whom English was not a first language, this feature of the local context shaped their navigation through healthcare services and their ability to make a claim for candidacy.

## Discussion

The conceptual framework of candidacy offers a counter to existing notions of healthcare utilisation, which have traditionally done little to elucidate the nature of access. In the MIA study we examined perceptions and understandings amongst South Asian parents with a child with asthma and explored their experiences of care, with a particular focus on identifying barriers to optimal care. Drawing on the candidacy framework, this paper has highlighted a number of areas in which barriers appear to inhibit successful asthma management.

Understanding asthma, including the recognition of symptoms and triggers, is crucial if families are to be able to assert a claim to candidacy and to achieve effective asthma management for their children. In this study, parents expressed confusion about the origins and symptoms of asthma and were often unfamiliar with biomedical definitions of the characteristics and management of the condition, a finding which has been demonstrated elsewhere [22, 23]. Parents were likely to describe receiving advice and guidance about their children’s health from members of their extended family, and though not all of this advice was taken, it added to the ambiguous context parents were negotiating. The significance of extended family perceptions may be of heightened importance within South Asian communities and it is important that health professionals are aware of and can respond to such perceptions in a sensitive manner if they conflict with traditional medical management. The wider cultural context of any particular family should therefore be acknowledged by healthcare professionals and understandings checked at the beginning and end of each consultation. It is important that ethnic differences in knowledge and beliefs about asthma, rather than being viewed as a ‘deficit’ in the knowledge base of South Asian families, are instead located within the specific conditions of the social context when information is provided, and educational interventions are enacted.

The BTS guidelines [15] recommend what information should be shared with patients with asthma, with SIGN and Asthma UK having developed leaflets that are available for healthcare professionals to provide to patients and families [24, 25]. Despite these resources being available and national recommendations instructing health professionals to take a role in providing education as part of their consultations and management plans, parents in this study reported such that information had not been provided or that they had difficulty in understanding the information. None of the parents recalled an asthma management plan being provided by any healthcare professional despite this being a national recommendation included as one of the national asthma quality standards [15]. Such plans need to be discussed with families in the light of their existing knowledge and interpretation of symptoms and actions, on an ongoing basis, not just once at diagnosis when recall may be difficult for parents if circumstances are stressful.

Uncertainty about symptoms and exacerbations affected parents’ ability to assert a claim to candidacy and their subsequent appearance at health services. Participants described a low level of awareness of asthma prior to diagnosis. Once diagnosed, without being able to assess severity, parents described feeling unable to know at what point and where to access healthcare for early intervention and this made them particularly vulnerable to adjudications about their child’s health since none had any formal means of assessing symptoms. No parents described having been provided with written asthma plans and no one reported employing any objective measure of severity, potentially exacerbating any deterioration in the child and the need to seek urgent care from the emergency department, and in some cases, to call an ambulance for help.

Accessing appropriate care is essential for ensuring a timely diagnosis, optimisation of treatment and the prevention and management of acute attacks. Previous experience of contact with the health service can influence patients’ future health-seeking behaviour [26] both positively and negatively. As with other chronic, long-term conditions, asthma management aims to keep the child functioning optimally, attending school and staying out of hospital [26]. Within the NHS, primary care services are available to initially provide a diagnosis of asthma and provide regular reviews to optimise management, with ‘out of hours’ services and emergency services being available for urgent care and acute severe attacks. However, emergency attendances for all clinical problems are increasing [27] with a higher attendance rate for South Asian children with asthma compared to White British families with a child with asthma. Drivers for this health-seeking behaviour need to be understood if South Asian families are to be encouraged and supported to manage their children’s asthma and to be made aware of the role of primary care services in relation to emergency care. In this study difficulties expressed by parents regarding the most appropriate place of presentation during an acute attack highlighted an important problem with current provision and with navigation through the NHS. The parents in this study chose (justifiably, it might be argued) to access ED if they were unable to access their GP, driven by previous experience when using the health system (e.g. being referred to ED during previous acute asthma attacks, or experiencing negative or delayed appointments with their GP) but also related to difficulties in recognition of severity of symptoms delaying timely access to treatment. Participants highlighted issues related to the consistency, timeliness and quality of the information provided which often led to confusion about the different medications available and the techniques required to deliver them, and practical barriers to accessing primary care in times of urgent need. Preventing ED admissions therefore requires improved communication of self-management strategies for families, provided in a culturally and linguistically appropriate manner. Health professionals need to continually assess parental understanding of health messages for this to be effective. 

Parents expressed feelings of being ‘fobbed off’ within the healthcare system and described delays in getting a diagnosis, which led to feelings of frustration or anger. Irrespective of waiting times at the ED, if parents had previously been sent to the emergency department via NHS Direct or the out of hours services, they would, in the next event, self-refer, bypassing other services and reinforcing a perception that hospital services could deliver more unequivocally expert knowledgeable care than general practice. Despite this, all parents described the need to use the GP as the first point of contact in a non-acute situation. This finding perhaps reflects a broader context in which there is no ‘gold standard’ test for asthma and a reluctance to diagnose asthma amongst healthcare professionals [28]. NICE (National Institute for Health and Care Excellence) are currently developing new guidelines on the diagnosis and management of asthma [29].

Many of the problems facing parents of a child with asthma are common to all communities: lack of understanding and knowledge about the origins or triggers of asthma; poorly communicated information; a lack of a written asthma plan; inconsistent care between healthcare professionals, and dissatisfaction with the health service [22, 23]. However, some issues such as prior knowledge and perceptions of asthma and poor information provision were exacerbated for some South Asian families, especially for those who experienced language barriers: a finding reported elsewhere in relation to candidacy in minority communities [30]. Furthermore, lay beliefs and membership of wider family and community networks may influence help-seeking behavior. This research demonstrates that a complex constellation of factors may mean that South Asian families are less well placed to successfully negotiate asthma services for their children. Recognition of this complexity and the multiplicity of help-seeking behavior can be aided by the use of the candidacy framework [31].

### Limitations

We use the term ‘South Asian’ to refer to members of communities in the UK who have their ancestral origins in India, Pakistan and Bangladesh. We recognise the limitations of this label and its potentially homogenising effects. In this study we sought to recruit a sample which was proportionally representative of South Asian groups: this was only partially achieved and means we have small numbers of some sub-groups within the broader South Asian category (i.e. those of Pakistani heritage). There is a mixture of interviews conducted in English and those conducted in a South Asian language and translated in to English.

## Conclusion

Our analyses using the candidacy conceptual framework demonstrate how a potential lack of alignment between the priorities and competencies of British South Asian families and the organization of health services combine to create vulnerabilities and difficulties in effectively managing childhood asthma. Thus there is a need for clear and clinically applicable interventions that have considered the various barriers and explanatory factors, including the impact of language differences. Using the candidacy framework in this context demonstrates the considerable efforts required from South Asian families in navigating and negotiating health services. Awareness of asthma and its aetiology was described as low by members of the communities studied, and in some cases perceptions appeared to differ from biomedical definitions and explanations. Further efforts therefore need to be made by healthcare professionals to raise awareness of symptoms and effectively communicate how, when and where to seek help for children. There is a clear need for consistent and effectively communicated information, especially regarding medication and improved diagnosis. Many South Asian parents in this study expressed confusion about the different types of medication prescribed and when and how to use them, and expressed significant serious concerns about side-effects, with several parents reporting taking independent decisions to increase or decrease their child’s use of medicines.

Several factors relating to the higher use of ED have been identified in this study. Parents themselves made several important suggestions for improving services, including: having presentations about asthma at easily accessible local community venues; parents being able to access an advice centre or telephone helpline to answer queries; having opportunities for sharing experiences with other families; having information provided in South Asian languages; longer GP appointments; providing follow-up appointments with asthma nurses; and providing more education for healthcare professionals to ensure consistency of care. Listening to South Asian parents through qualitative interviews has demonstrated multiple levels of influence on health outcomes at the patient, provider and health systems levels. Deficit models of culture in minority ethnic groups as an explanation for health inequalities are inadequate and a shift is needed to recognise and address the responsibility of healthcare systems to develop services which are sensitive and appropriate to the needs of their communities.

## Abbreviations

BTS, British Thoracic Society; ED, emergency department; GP, general practitioner; MIA, management and interventions for asthma study; NHS, National Health Service; NICE, National Institute for Health and Care Excellence

## Footnotes

1 ‘Secondary carers’ were family members, such as grandparents, aunts or uncles, involved in a child’s care.

2 The British Thoracic Society guideline on the management of asthma in children is used to refer to the severity of a child’s asthma and the level of treatment they require. Level 1 is the least severe and level 5 the most.

3 The data from the children are reported elsewhere (see [12]).
